# Effects of fat-to-sugar ratio in excess dietary energy on lipid abnormalities: a 7-month prospective feeding study in adult cynomolgus monkeys

**DOI:** 10.1186/s12944-018-0950-y

**Published:** 2019-01-05

**Authors:** Ke-Wei Wang, Bai-Quan Xiao, Bi-Hai Li, Yi-Yan Liu, Zhi-Yuan Wei, Jun-Hua Rao, Jian-Huan Chen

**Affiliations:** 1 Department for hospital infection, Affiliated Hospital of Jiangnan University (Wuxi Third People’s Hospital), Wuxi, 214041 Jiangsu China; 2 Laboratory of Genomic and Precision Medicine, Wuxi School of Medicine, Jiangnan University, 1800 Lihu Avenue, Wuxi, 214122 Jiangsu China; 3 Joint Primate Research Center for Chronic Diseases, Jiangnan University and Guangdong Institute of Applied Biological Resources, Jiangnan University, No 1800 Lihu Avenue, Wuxi, Jiangsu, Wuxi, 214122 Jiangsu China; 4 School of Biotechnology, Jiangnan University, 1800 Lihu Avenue, Wuxi, Jiangsu China; 5 Guangdong Key Laboratory of Animal Conservation and Resource Utilization, 105 Xingang Rd. West, Guangzhou, 510260 Guangdong China; 6 Guangdong Public Laboratory of Wild Animal Conservation and Utilization, 105 Xingang Rd. West, Guangzhou, Guangdong China; 7 Guangdong Institute of Applied Biological Resources, 105 Xingang Rd. West, Guangzhou, Guangdong China

**Keywords:** High-sugar diet, High-fat diet, Lipid abnormality, Fasting glucose, Cynomolgus monkey

## Abstract

**Background:**

Excess energy intake contributes to metabolic disorders. However, the relationship between excess sugar and fat in their contributions to metabolic abnormalities remains to be further elucidated. Here we conducted a prospective feeding experiment to evaluate effects of dietary fat-to-sugar ratio on diet-induced metabolic abnormalities in adult cynomolgus monkeys.

**Methods:**

Four groups of adult cynomolgus monkeys were fed regular chow plus emulsion with combinations of high sugar (HS) or low sugar (HS) and low fat (LF) or high fat (HF) for 7 months. Plasma levels of total cholesterol (TC), low-density lipoprotein cholesterol (LDL-C), high-density lipoprotein cholesterol (HDL-C), triglyceride (TG) and blood glucose were measured for all the four groups of animals during the experiment.

**Results:**

Plasma levels of TC and LDL-C gradually increased in all 4 diets groups, with the highest increase found in the LSHF group compared to the other three groups (*P* = 0.0018 and *P* = 0.0005 respectively). HF induced increased fasting glucose (*P* = 0.0077) and HS induced higher TG (*P* = 0.0227) respectively. Intriguingly, HSHF led to dramatically smaller magnitude of increase in LDL-C and TC levels compared to LSHF, while such difference was absent between the LSLF and LSHF groups. Our findings thus indicate interactive effects of HS and HF on TC and LDL-C. In addition, HF exhibited stronger effects on lipid abnormalities than HS.

**Conclusions:**

In the current study, our prospective feeding experiment in adult cynomolgus monkeys revealed effects of different fat-to-sugar ratios on diet-induced metabolic abnormalities. Furthermore, our findings suggest that not only excess dietary energy but also the balance of dietary fat-to-sugar ratio matters in diet-induced lipid abnormalities.

## Introduction

Metabolic abnormalities are associated with various clinical conditions and diseases. Metabolic syndrome (MS) is generally considered as a prerequisite for cardiovascular diseases (CVD), with a direct relationship with increased risk of CVD [1]. Excess energy intake from dietary consumption can have a strong potential impact on metabolic disorders and related diseases. Epidemiological studies have suggested that high-sugar (HS) diet and high-fat (HF) diet are associated with MS, CVD and other related diseases [2–4].

However, the relationship between excess dietary sugar and fat in their contributions to metabolic abnormalities and related diseases remains controversial. A cohort study suggested that low carbohydrate diets had beneficial effects on metabolic risk factors, and these effects were comparable to those observed in low-fat (LF) diet [5]. In contrast, some studies have suggested that decreased fat intake and increase in carbohydrate might contribute to lower cardiometabolic risk profiles [6]. In addition to epidemiological studies, animal studies also reported inconsistent findings − effects of different diets on metabolic abnormalities, most of which were conducted in rodents. Yoo et al found that rats fed with a HS diet exhibited higher total triglyceride (TG) levels compared to a regular diet, while Zaman et al observed no difference in cholesterol concentrations between rats fed with a HS diet and controls [7, 8]. However, another study demonstrated that increased fat-to-carbohydrate ratio in diets led to insulin resistance and increased LDL-C in rats [9]. Differences in lipid metabolism between animal species and humans, especially between rodents and primates might contribute to such inconsistency. In addition, long-term prospective feeding experiments are needed to delinear the relationship between excess dietary sugar and fat in metabolic abnormalities.

By far, there have been limited data on prospective feeding experiments that evaluate effects of different ratio of excess dietary fat and sugar on diet-induced metabolic abnormalities in nonhuman primates, which are supposed to closely resemble their human counterparts. Therefore, we conducted a prospective feeding experiment among adult cynomolgus monkeys to determine effects of diets with different fat-to-sugar ratios in excess dietary energy intake. The current study provides new insight into the relationship between excess sugar and excess fat in diet-induced lipid abnormalities in primates.

## Materials and methods

### Animals

A total of 23 adult (8–20 years old) male cynomolgus monkeys were obtained from Blue Bird Zhongke Laboratory Animals Co. Ltd. (Guangzhou, China), which is accredited by the Association for the Assessment and Accreditation of Laboratory Animal Care International (AAALAC). All of the animals were confirmed to be in healthy conditions by records and veterinary examination before the experiment.

The study complied with protocols approved by the Animal Ethics Committees of Jiangnan University and were in compliance with the Guide for the Care and Use of Laboratory Animals (National Research Council (US) [10].

### Dietary feeding

During the experiment, all monkeys were fed normal chow (a grain-based meal, 3.08 kcal/g) at a dose of 30 g/kg per day (Table 1). In addition, all monkeys were provided with fresh fruit and vegetable once daily and fresh water ad libitum. From the beginning of the experiment, the monkeys were divided into four diets groups, and were fed normal chow daily plus emulsion with different 2 × 2 combinations of excess sugar and fat six days a week: HSHF (*N* = 6), HSLF (N = 6), LSHF (N = 6), and LSLF (N = 6) (Table 2). The factorial design allows us to assess the possible interaction between excess sugar and fat in diets. All animals were fed these diets for 7 months.

**Table 1:** The percentage by weight of compositions in the regular chow

Chow diet	Weight (%)
Protein	22.2
Fat	6.9
Carbohydrate	49.0
Crude ash	6.5
Lysine	0.8
Calcium	1.1
Water and other volatile products	9.7
Coarse fiber	3.7

**Table 2:** Composition of emulsion with excess sugar and fat in the four groups

	HSHF (kcal/ml)	HSLF (kcal/ml)	LSHF (kcal/ml)	LSLF (kcal/ml)
Excess sugar (sucrose)	1.55	1.55	0.77	0.77
Excess fat (lard)	3.60	1.80	3.60	1.80
Total caloric intake / ml	5.15 kcal/ml	3.35 kcal/ ml	4.37 kcal/ ml	2.57 kcal/ml

*HS/HF* High-sugar/high-fat, HS/LF, High-sugar/low-fat, *LS/HF* Low-sugar/high-fat, *LS/LF* Low-sugar/low-fat

In addition to a daily regular chow, monkeys in the four groups were fed excess sugar and fat described above at a dose of 5 ml/kg of body weight six days per week

### Blood sample collection and testing

Blood samples were collected at the beginning of the study (month 0) for the baseline group, and at month 1, 3, 5 and 7 for the four different diets groups. Plasma was stored at − 80 °C before used to measure Plasma concentrations of TG, TC, HDL-C and LDL-C using an ACE clinical analyzer (Alfa Wasserman Diagnostic Technologies, LLC, West Caldwell, NJ).

### Statistical analysis

Data are presented as means ± standard error of the mean, unless specified otherwise. To evaluate impact of the four different diets on body weight, fasting glucose, TC, TG, HDL-C and LDL-C levels, time (month) was considered as a continuous covariate in the mixed linear effect model. To further estimate whether interaction between sugar and fat is effective at the body weight gain, increased fasting glucose and lipid abnormalities at different time points, a time×sugar×fat-factor repeated measure factorial design was applied. If significant, post hoc tests (Turkey) were performed for individual group differences. Statistical significance was set at *P* < 0.05. Statistical analysis was performed using SAS version 9.2 (SAS Institute Inc., Cary, NC, USA).

## Results

**Fig. 1:**
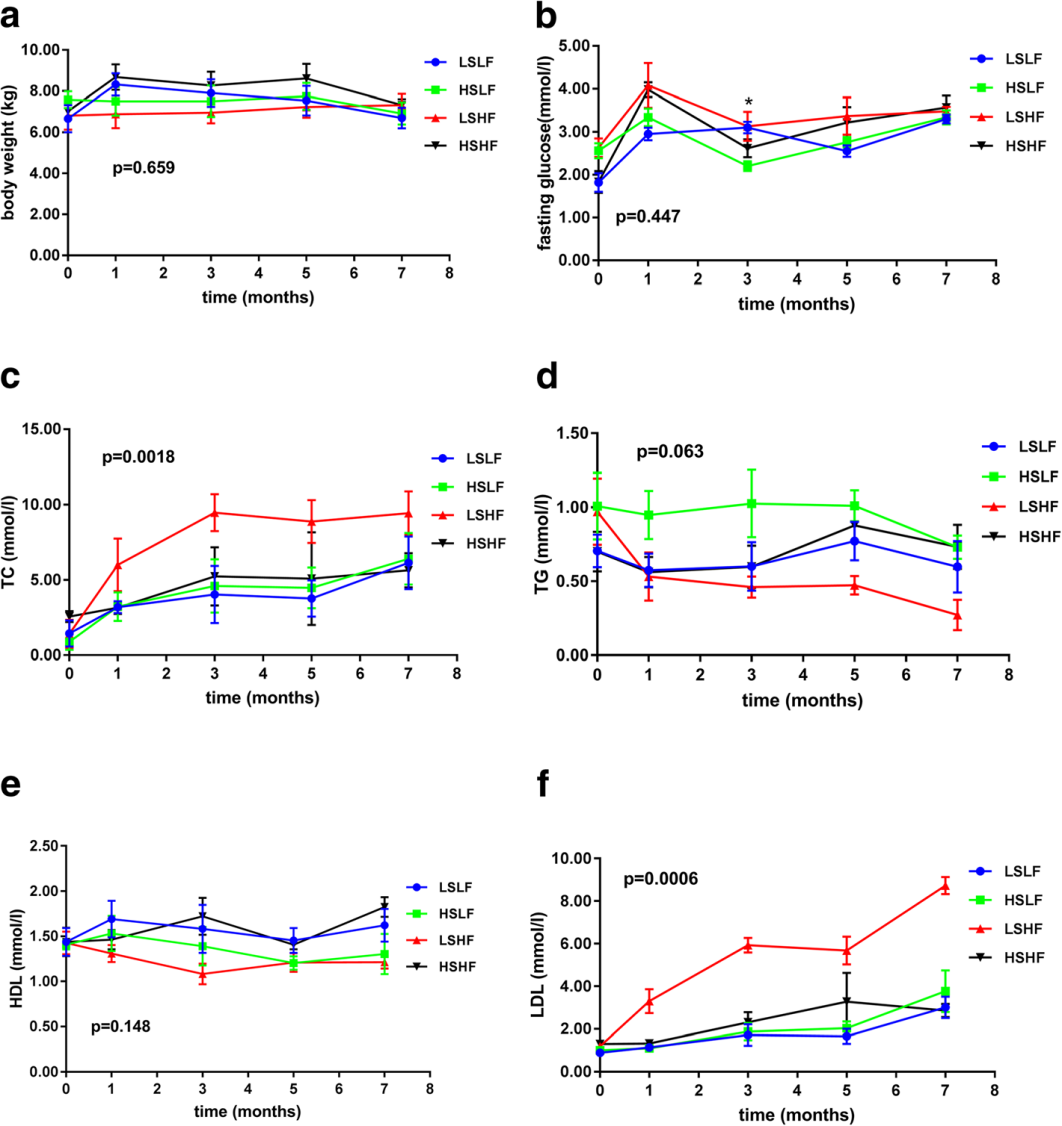
Mean changes in body weight, fasting glucose and lipid profiles among four groups with different ratio of excess fat and sugar throughout the 7-month intervention. The monkeys in the four groups were fed regular chow plus emulsion with different ratio of excess fat and sugar. Results are shown for body weight (**a**), fasting glucose (**b**), total cholesterol (TC) **c**), triglyceride (TG) (**d**), HDL-C (**e**), LDL-C (**f**). Data are based on mixed-model analysis of variance. The *P* value at the upper or lower left indicates the test of whether the change between baseline and intervention period (mean of every two months) differed significantly between cynomolgus monkeys assigned to four groups. The *P* values for the comparison between the LSLF group and the HSLF group are 0.733 for total cholesterol and 0.934 for LDL-C cholesterol. The *P* values for the comparison between the LSLF group and the LSHF group are 0.0001 for TC and 0.0001 for LDL. The *P* values for the comparison between the HS/LF group and the HSHF group are 0.898 for TC and 0.885 for LDL. The *P* values for the comparison between the LS/HF group and the HSHF group are 0.0001 for total cholesterol and 0.0001 for LDL-C cholesterol. *p < 0.05

Body weight, fasting glucose, TC, TG, HDL-C and LDL-C levels in the four diets groups are shown in Fig. 1a, b, c, d, e and f. The mixed linear effects models contained body weight, fasting glucose, TC, TG, HDL-C and LDL-C levels as response variable, and time (month) as a continuous covariate to allow the slope of the regression line representing the change in outcome over time to be assessed. TC and LDL-C increased throughout all four diets groups, with the highest increase in the LSHF group compared with the other three groups (*P* = 0.0018 and 0.0006, respectively). Body weight, fasting glucose, TG, or HDL-C showed no significant difference among all groups (*P* = 0.6586, 0.4467, 0.0628 and 0.1483, respectively).

**Fig. 2:**
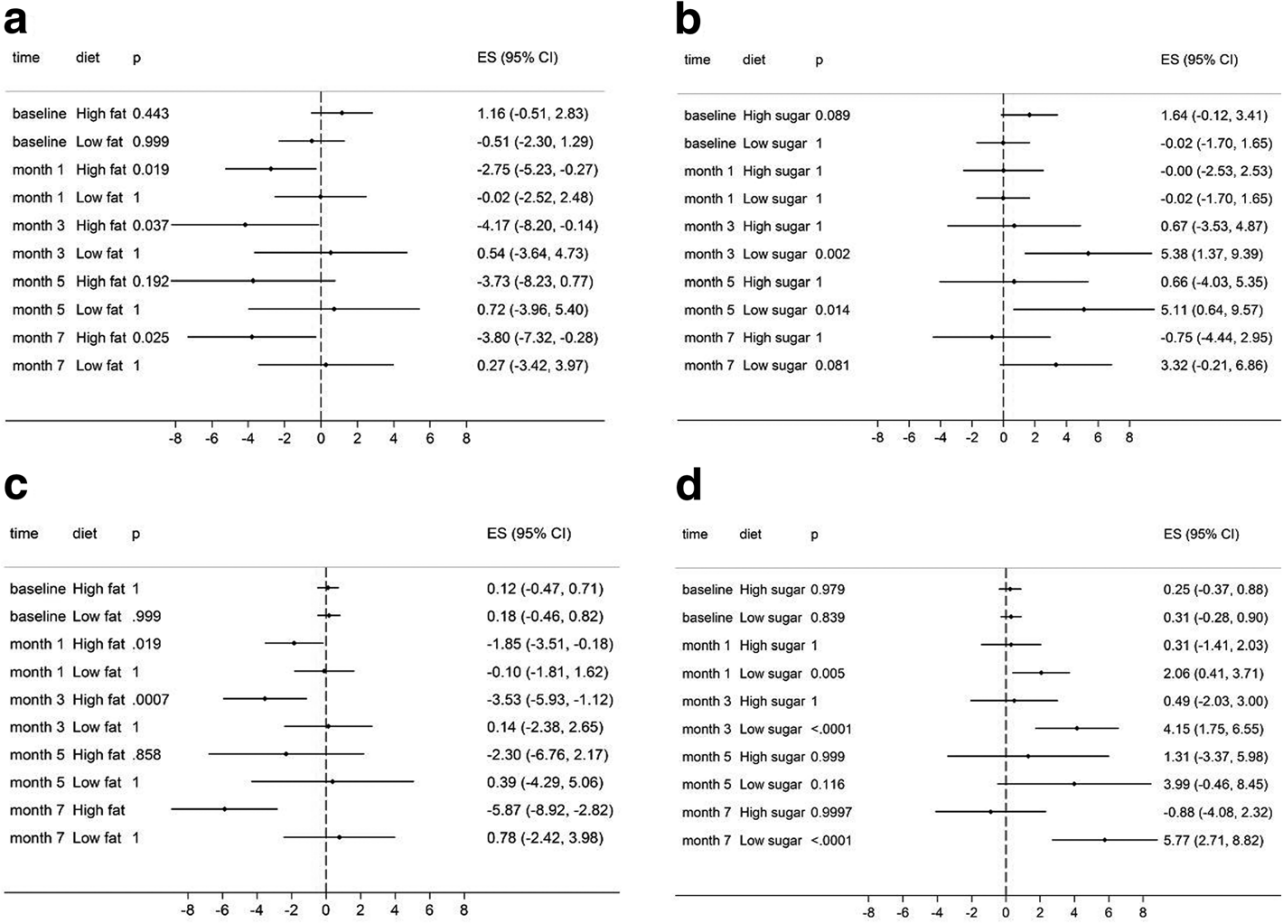
Forest plots showing excess sugar×fat interaction model estimates by five time points. ES stand for adjusted mean differences after analysis of linear mixed effects models adjusting body weight by time. Panel (**a**) shows mean differences of TC between HS and LS combined with HF or LF, Panel (**b**) shows mean differences of TC between HF and LF combined with HS or LS, Panel (**c**) shows mean differences of LDL-C between HS and LS combined with HF or LF, Panel (**d**) shows mean differences of LDL-C between HF and LF combined with HS or LS

We next determined whether HS or HF could be significantly associated with body weight, fasting glucose, TC, TG, HDL-C and LDL-C levels at each time point. HS and HF were not significantly associated with body weight and HDL-C (all *P* > 0.05) while HS contributed to a significant increase in TG (*P* = 0.0227). Two significant sugar×fat×time interactions for TC and LDL-C were observed (*P* = 0.0005, *p* < 0.0001). At month 1, month 3, month 5 and month 7, under a HF condition, the mean differences in TC in LS compared that in HS were − 2.75 (95% CI -5.23 to − 0.27; *P* = 0.019), − 4.17 (95% CI -8.20 to − 0.14; *P* = 0.037), − 3.37(95% CI -8.23 to − 0.77; *P* = 0.192) and − 3.80 (95% CI -7.32 to − 0.28; *P* = 0.025) respectively (Fig. 2a). However, under a LF condition no significant difference in TC were found between HS and LS at all four time points (all *P* > 0.05). Likewise, under a LS condition, the mean differences in TC in HF compared that in LF were − 0.02 (95% CI -1.70 to 1.65; *P* = 1), 5.38 (95% CI 1.37 to 9.39; *P* = 0.002); 5.11 (95% CI 0.64 to 9.57; *P* = 0.014) and 3.22 (95% CI -0.21 to 6.86; *P* = 0.081) respectively (Fig. 2b). In contrast, under a HS condition, at all four time points TC showed no significant difference between LF and HF (*P* > 0.05). Similarly, compared with LS, HS contributed to decreased LDL-C levels under a HF condition, which in contrast was absent under a LF condition. Likewise, compared with LF, HF increased LDL-C levels under a LS condition, which was not found under a HS condition (Fig. 2c and d).

The overall effect of HS or HF across 4 time points was further assessed. Though there were no significant differences in fasting glucose throughout the four diets groups for all monkeys, 2 × 2 factorial analysis showed a significant overall increasing trend of fasting glucose over time (*P* < 0.0001). Only HF contributed to a significant increase in fasting glucose after adjusted for body weight (*P* = 0.0077).

## Discussion

Cynomolgus monkeys, similar to humans, are susceptible to age-related chronic metabolic abnormalities and related diseases, such as CVDs, MS, dyslipidaemia and type 2 diabetes [11–13]. In current study, we used a 2 × 2 factorial, 7-month prospective feeding experiment to evaluate effects of excess sugar and fat on metabolic changes and their relationship in adult cynomolgus monkeys. Our finding showed an interactive effect between excess sugar/fat in diet-induced abnormal TC and LDL-C levels.

Abnormal TC and LDL-C levels that increased over time were observed in diets with different compositions of excess sugar and fat in the current study. A previous study found increase over time in concentrations of TC and LDL-C in baboons exposed to a HS/HF diet for 8 weeks [14]. Suzuki reported that, concentrations of TC and LDL-C increased with HS/HF diet feeding time (up to 28 weeks) in cynomolgus monkeys [15]. Similar results were found in our current study. As shown in Fig. 1 that the all of the four groups had overall increasing concentrations of TC and LDL-C over time. Nevertheless, the LSHF group have the highest concentrations of TC and LDL-C among four feeding diets. In accordance with such findings, a previous study in rats showed that fats assigned to a high-energy diet with 9% carbohydrate have higher concentrations of TC and LDL-C than those assigned to a high-energy diet with 30% carbohydrate [9]. Other study also showed that the concentration of TC in rats exposed to a HS (fructose)/ HF diet for 8-week experimental diet was lower than those exposed to a HF diet despite showing no significant difference [7]. Interestingly, a recent cross-sectional study examining the associations between dietary carbohydrates and fats and lipid abnormalities among 14,301 adults of Korean adults found that percentage of energy from carbohydrate was inversely associated with elevated TC and elevated LDL-C, while high dietary fat intakes were positively associated with elevated TC and LDL-C [16]. Therefore, our data suggested an interactive effect of HS diet and HF diet on concentrations of TC and LDL-C in adults. In this study, mixed linear effects model analysis showed that HS might reduce the effect of HF in diet-induced elevated TC and elevated LDL-C at month 3, 5 and 7, respectively. Though HS diet and HF diet are usually defined by their reciprocal relationship [16], the underlying mechanism of such interaction between HS and HF in diets inducing elevated TC and LDL-C remains unclear, and further study was thus warranted. Our results suggested that fat reduction rather than sugar restriction may be recommended as a potential nutritional approach to control blood TC and LDL-C concentration.

In the current study, mixed linear model analysis of HS and/or HF in diet-induced HDL-C and TG showed that HS might only lead to a significant increase in TG, but not in HDL. Inconsistent responses on TG concentration which have been reported in rats fed with HS diet in previous studies. Rats exposed to a high fructose diet showed a significant increased TG levels compared to the other exposed to a regular diet [7, 17, 18]. In contrast, other studies have reported no change in TG concentration in rats fed with high fructose diet for a short and long period [8, 19, 20]. In contrast our results in cynomolgus monkeys were similar to findings that have been reported in humans. Welsh JA et al found that increased added sugars are associated with elevated TG among US adults [3]. Stanhope KL et al investigated 187 young adults in USA and found that consuming beverages containing 10, 17.5%, or 25% energy requirements from high-fructose corn syrup produced dose-dependent increases in circulating concentrations of postprandial TG [4]. Previous a meta-analysis of randomized controlled trials in humans provides evidence that higher compared with lower intake of sugars are positively associated with increased concentrations of TG [21]. In addition, TG increases with age in cynomolgus monkeys, which resembles findings that have been reported in human adult populations [22]. Therefore, we conclude that compared to rodent models, our nonhuman primate (NHP) model with adult cynomolgus monkeys could be more useful and relevant to investigate TG levels induced by excess sugar intake.

## Conclusions

In the current study we conducted a prospective feeding experiment of adult cynomolgus monkeys exposed to high-energy diets of different fat-to-sugar ratios. Our NHP results of excess energy intake demonstrated that, although HS and/or HF lead to lipid abnormalities and increased fasting glucose, interactive effects of HS and HF on TC and LDL-C were observed, and the effect induced by HF on metabolic risk factors is generally stronger than that by HS. Our finding thus suggested that not only excess dietary energy but also the balance of dietary fat-to-sugar ratio matters in nutritional intervention to reduce risk factors of lipid abnormalities and its related diseases.

## Abbreviations

AAALAC: Association for the Assessment and Accreditation of Laboratory Animal Care International
CVD: Cardiovascular diseases
HDL-C: high-density lipoprotein cholesterol
HF: High-fat
HS: High-sugar
LDL-C: Low-density lipoprotein cholesterol
MS: Metabolic syndrome
NHP: Nonhuman primate
TC: Total cholesterol
TG: Triglyceride
